# Cortical latency predicts reading fluency from late childhood to early adolescence

**DOI:** 10.1016/j.dcn.2025.101616

**Published:** 2025-10-22

**Authors:** Fang Wang, Quynh Trang H. Nguyen, Blair Kaneshiro, Anthony M. Norcia, Bruce D. McCandliss

**Affiliations:** a Graduate School of Education, Stanford University, Stanford, CA, USA; b Department of Psychology, Stanford University, Stanford, CA, USA; c Wu Tsai Neurosciences Institute, Stanford, CA, USA

**Keywords:** EEG, SSVEP, N170, Reliable Components Analysis (RCA), Visual Word Recognition, Latency, Typical developing children

## Abstract

Progressive development of reading comprehension fluency from late childhood to early adolescence is remarkably linked to changes in the temporal dynamics of visual word recognition. EEG/ERP based measures of how an individual participant’s cortical timing for visual word recognition change over development are limited by low reliability. We present a novel approach to this challenge that individually models cortical latency to visual word forms by extracting phase values from Steady-State Visual Evoked Potentials (SSVEPs) for each participant. The resulting precise and reliable timing information for neural signatures underlying visual word form processes help account for the development of fluent reading comprehension. Typically developing readers (n=68), aged 8–15 years, viewed streams of four-character stimuli presented at 3 Hz, which evoked large significant power spikes from every participant. Linear phase by frequency functions across harmonics at 3, 6, and 9 Hz were consistent with a delay model, indicating a mean latency of 170 ms. Subject-level latencies revealed (a) high internal consistency (r=.94); (b) stability across variations in character-level (letters, unfamiliar pseudo-characters) and word-form level (words, nonwords, pseudofont strings) manipulations; (c) a linear relationship with age; and most remarkably, (d) a strong relationship with individual variation in the fluency of reading comprehension, that was (e) mediated by word naming speed. Results suggest a promising new approach for investigating the neural basis of reading development across several levels of processes, with temporal precision at the individual level that holds translational significance for promoting population-level fluency in reading comprehension.

## Graphical abstract

**Figure:**
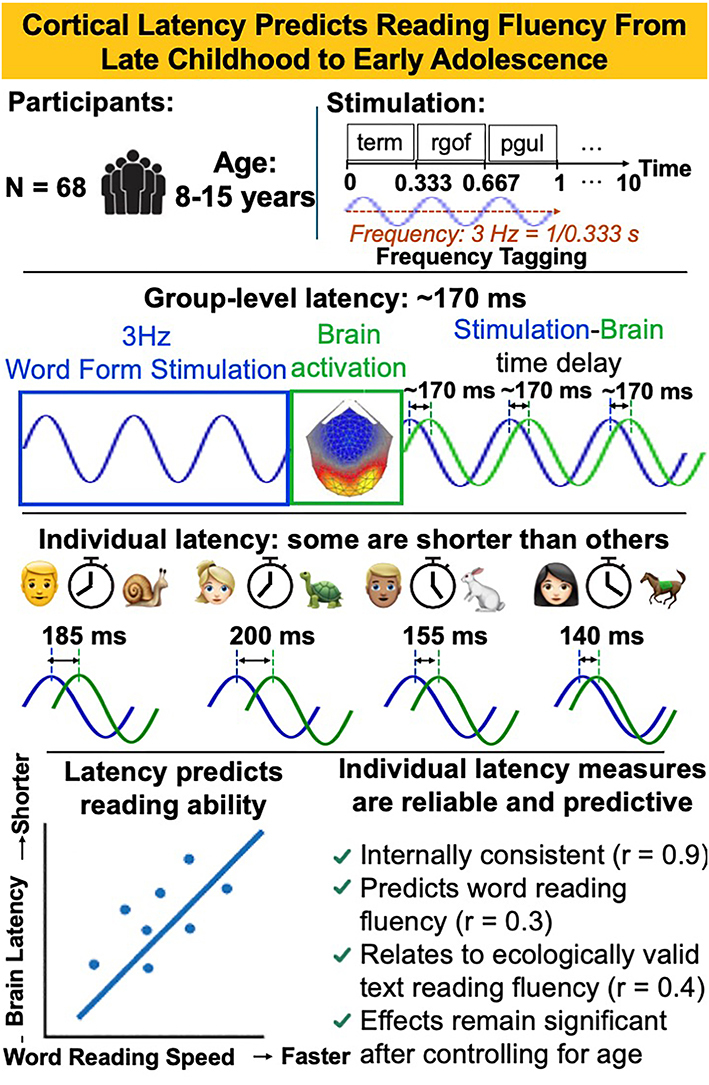

## 1 Introduction

The emerging field of developmental cognitive neuroscience aims to understand how complex psychological functions arise from more elementary cognitive operations by integrating behavioral and neural approaches. Reading development offers a paradigmatic example: decades of work linking behavioral and neural measures of visual word recognition have transformed our understanding of how this elementary operation supports the complex skill of fluent reading comprehension (McCandliss, 2010).

From late childhood to adolescence, many youth experience a shift in reading from a slow, effortful process of decoding words and parsing sentences to a fluent, engaging activity that supports social, emotional, and intellectual growth. This rise in reading comprehension fluency reflects progressive changes in the temporal dynamics of reading processes that eventually approach the speed of oral language comprehension (Brysbaert, 2019). Yet, research on this developmental stage remains limited (Khanolainen et al., 2024), with existing studies pointing to diverse developmental trajectories and strong links between higher-order comprehension fluency and the quality of underlying word recognition processes.

The development of reading fluency is widely recognized as the bridge from early decoding accuracy to skilled reading comprehension (Pikulski and Chard, 2005). Changes in the temporal dynamics of reading skills are thought to play a pivotal role in both reading comprehension and overall literacy development (Yildiz et al., 2014). Once children acquire accurate word decoding skills in early grades (e.g., second grade), continued fluency development from late childhood to early adolescence becomes critical for advancing toward proficient, meaning-focused reading (Chard et al., 2002). Conversely, persistent challenges in developing reading fluency have been linked to difficulties in understanding written texts (Álvarez-Cañizo et al., 2015) and broader academic underachievement (Bigozzi et al., 2017). As a result, measures of reading fluency and its development have become central to understanding individual differences in reading, including individuals with developmental dyslexia.

While a host of factors are thought to influence reading fluency across individuals (Pennington, 2006), word-recognition speed stands out as a consistently dominant predictor of reading ability (Landerl and Wimmer, 2008). Several studies have shown that skilled and poor readers differ not so much in word-recognition accuracy but rather in word-recognition speed (Karageorgos et al., 2020, Ziegler et al., 2003).

Word-recognition speed has typically been assessed through behavioral measures such as reaction times in tasks like lexical decision (Karageorgos et al., 2020). While informative, these behavioral measures may not fully capture the underlying temporal dynamics of word recognition. In contrast, neural timing markers provide a more direct and fine-grained index of the temporal processes involved, offering insights beyond what is available from behavioral response times alone (Maurer and McCandliss, 2007). Therefore, examining the neuro-temporal dynamics underlying visual word recognition is crucial for a robust understanding of childhood reading fluency development.

Electroencephalography (EEG) holds promise for capturing the neural-temporal dynamics of visual word recognition processes with millisecond-level precision (Posner and McCandliss, 1993). Numerous studies using Event-Related Potential (ERP) paradigms have characterized the N170 (also sometimes labelled N1) component — a left-lateralized negative deflection over occipito-temporal regions that typically peaks between 150 and 200 ms after stimulus onset — during visual word recognition (Maurer et al., 2005, Ruz et al., 2005, Wang and Maurer, 2017, Yoncheva et al., 2010, Yoncheva et al., 2015). By examining the latency (i.e., the timing delay of the neural response relative to stimulus onset) of the N170 component during word processing, researchers have gained valuable insights into the temporal dynamics of reading development. For instance, visual word form N170 peak latency has been shown to decrease from 2nd to 5th grade, reflecting increasing efficiency in visual word processing with reading experience and maturation (Maurer et al., 2011). Some studies have also reported group differences in N170 latency between individuals with dyslexia and typically developing readers, suggesting delayed or disrupted early visual word processing in dyslexia (Hämäläinen et al., 2011, Spironelli et al., 2010). However, other studies have found comparable latencies between typically developing children and those with developmental reading difficulties or poor reading (Kast et al., 2010, Hasko et al., 2013, Zhao et al., 2014), indicating that group differences in N170 latency may not be consistently observed across studies or tasks. Moreover, N170 latency appears to be insensitive to individual differences in reading fluency development (Amora et al., 2022, Brem et al., 2009). This insensitivity may be due to trial-to-trial latency jitter and variability across individuals, which makes it challenging to obtain reliable latency estimates at the individual level from transient ERP signals (Smulders, 2010).

Steady-State Visual Evoked Potential (SSVEP) paradigms, in which a sequence of stimuli are presented at a predefined periodic rate (e.g., 3 Hz), elicit neural responses at the stimulation frequency and its harmonics (i.e., integer multiples of the stimulation frequency; Norcia et al., 2015). These responses are referred to as SSVEPs due to their stability in both amplitude and phase over time, making them a useful complement to ERP paradigms. SSVEPs offer robust and high signal-to-noise ratio (SNR) responses in nearly every subject with as few as 120 seconds of stimulation per condition (Wang et al., 2023), and have been linked to reading development (van de Walle de Ghelcke et al., 2021, Wang et al., 2024). Because of their robustness and reduced sensitivity to trial-by-trial variability, SSVEP paradigms are well-suited for individual-level temporal measurements (Norcia et al., 2015, Wang et al., 2021). Importantly, SSVEP studies have identified a neural source that aligns with the well-established ERP N170 component, showing amplitude modulations in response to effects previously attributed to the N170—including print tuning (ERP: Maurer et al., 2005; SSVEP: Lochy et al., 2016), sensitivity to orthographic regularity (ERP: Hauk et al., 2006; SSVEP: Wang et al., 2023, word frequency (ERP: Hauk et al., 2006; SSVEP: Wang et al., 2024, Wang et al., 2025), and learning of novel words (ERP: McCandliss et al., 1997; SSVEP: Wang et al., 2024, Wang et al., 2025).

More importantly, the phase of SSVEP signals has proven to be highly informative in studies of the precise timing of neural processes. In 1970, Da Silva and colleagues calculated what has come to be called “apparent latency” of early cortical processes for vision by examining the slope of the *phase-vs-stimulus temporal frequency* function after having presented the same stimulus condition at multiple temporal frequencies (Da Silva et al., 1970). When the phase-by-frequency plot is linear over a substantial range, the apparent latency can be calculated using the following formula:

d=1/360×δφ/δf

Here, *d* is the apparent latency in seconds, δφ is the change in phase over the frequency range in degrees, and δf is the change in frequency in Hz. δφ/δf is the slope of the phase-by-frequency plot.

Yeatman and Norcia extended this phase-by-frequency latency method to study the temporal dynamics of word-selective SSVEP responses (Yeatman and Norcia, 2016). They alternated full-screen images of scrambled and intact text across a range of frequencies from 1–12 Hz. Calculating the slope across these stimulation frequencies revealed early (141–160 ms) and late (>250 ms) left lateralized text-selective responses at group-level.

Presenting a single stimulus condition at multiple frequencies, as in Yeatman and Norcia (2016), is both time consuming in general and arduous in particular for children. Fortunately, a more efficient method has recently been validated, taking advantage of SSVEP paradigms that elicit multiple harmonics of the stimulus frequency, thereby providing the necessary frequency range to model latencies using the same procedures. Norcia and collegues noted that the phase of the SSVEPs to contrast-modulated luminance probes increased linearly over the first several harmonics of the stimulation frequency (Norcia et al., 2020). This linear function was then used to derive the latency for the SSVEPs using the *phase-by-harmonic* slope that emerged when results were averaged across the group of participants (Norcia et al., 2020). The assumption underlying this approach is that the harmonics of the group-level evoked response come from a single cortical source with a fixed delay. The *phase-by-harmonic* approach has since been successfully applied in recent SSVEP studies of word recognition, yielding robust group-level latency estimates in both adults and children (Wang et al., 2021, Wang et al., 2023). For example, Wang et al. (2021) reported group-level activation over the left ventral occipitotemporal area with a latency of approximately 170 ms when comparing words to pseudofonts processing in adults (Wang et al., 2021), while in children this response occurs later (Wang et al., 2023). Leveraging the high SNR and phase stability of SSVEPs, the current study was designed to validate and extend this approach by fitting phase-by-harmonic slopes to multiple harmonics at the single-subject level.

Of note, most prior SSVEP work has used the “oddball” paradigm—where a sequence of *base* stimuli (e.g., pseudofont strings) are periodically replaced by contrastive *oddballs* (e.g., words) (Lochy et al., 2015). By manipulating the contrast between base and oddball stimuli — such as words vs. pseudofonts, words vs. nonwords, or nonwords vs. pseudofonts — researchers can isolate neural responses associated with different levels of processing. Specifically, SSVEP responses emerging at the slower oddball frequencies (e.g., 1 Hz) are thought to reflect categorical discriminations related to higher-level visual processes such as visual word recognition, while the responses at the faster base frequencies (e.g., 3 Hz) are often interpreted as only reflecting lower-level sensory processes that are relatively insensitive to visual word form processing and stimulus contrasts (Lochy et al., 2016, Volfart et al., 2021). Even in one of our own studies, which used the same stimuli as the current experiment, no condition effects were observed for base amplitude (Wang et al., 2021). However, recent SSVEP studies using a 3 Hz base stimulation rate in children have revealed that multiple neural sources underlie the base response, some of which are consistent with higher-level visual word processing in regions such as the *visual word form area* (VWFA) (Wang et al., 2023, Wang et al., 2024). Such results highlight the importance of analyzing base responses in greater detail — particularly their temporal dynamics — as this could enhance our understanding of the neural basis of both general visual and word-related processes and their relationship with reading ability, potentially informing future research in this area.

Examination of the temporal dynamics of the base response is also merited by recent converging evidence suggesting that many aspects of developmental challenges in reading are linked to subtle temporal processing deficits (Stein, 2023). For example, McLean et al. (2011) found that word-recognition speed is a unique and significant predictor of reading ability, independent of phonological processing or domain-general cognitive skills. Consequently, the current study examines the temporal dynamics of the *base* responses using the phase-by-harmonic function validated in several recent studies (Norcia et al., 2020, Wang et al., 2021, Wang et al., 2023, Wang et al., 2024) to derive cortical latencies of visual word form processes at the individual-level. Based on previous findings showing shorter latencies in adults than in children (Wang et al., 2021, Wang et al., 2023), we hypothesized that shorter cortical latency would be associated with both increases in age and higher reading fluency, highlighting its significant role in the development of fluent reading comprehension from late childhood to early adolescence. Furthermore, as reading fluency bridges decoding and comprehension, we predicted that the relationship between cortical latency (milliseconds) and reading comprehension (sentences per minute) would be mediated by the speed of visual word recognition. Critically, this study goes beyond modeling the central tendencies of group-level SSVEP responses and also derives individual subject-level responses, which enable a rigorous examination of the connection between these cortical latencies and measures of reading skills in children at multiple levels of analysis.

## 2 Methods

### 2.1 Participants

Data from 68 typically developing, native English-speaking children (3rd–8th grade, between 8 and 15 years old, m = 10.8 years, sd = 1.8 years, 36 females) were analyzed. Data from three additional children were collected but not analyzed here due to EEG data-quality issues (i.e., excessive movement during recording, incomplete EEG data, n = 2) or visual impairment (cataract, n = 1). All participants had normal or corrected-to-normal visual acuity and had no diagnosed reading or learning disabilities. Reading proficiency, as measured by standardized assessments (see Section 2.2 Behavioral Assessments), varied across participants, allowing for examination of individual differences. After the study, each participant received a small toy for their time.

### 2.2 Behavioral assessments

Each participant completed a 30-min behavioral assessment either before or after the EEG session. All children were tested for handedness (Edinburgh Handedness Inventory, Oldfield (1971)), silent reading efficiency (i.e., speed and accuracy) and comprehension fluency (Test of Silent Reading Efficiency and Comprehension, TOSREC, Wagner et al., 2010), word reading efficiency (Test of Word Reading Efficiency, Second Edition, TOWRE-2, Torgesen et al., 2012), and non-verbal intelligence/fluid reasoning (the Matrices subtest of the Kaufman Brief Intelligence Test, Second Edition Revised, KBIT-2, Kaufman, 1990). Intelligence tests are typically included in dyslexia studies to ensure any performance deficits are not explained by more general cognitive challenges (Freed et al., 2017), and included here to allow a clearer interpretation of the relationship between neural temporal dynamics and reading outcomes. Results of the behavioral assessments are summarized in Table 1.

**Table 1:** Performance on behavioral assessments. Values are range and mean(±SD). TOSREC: Number of sentences read and correctly verified within three minutes. TOWRE: Number of real words and pronounceable nonwords read in 45 s. KBIT: Number of correct responses on the nonverbal reasoning subtest.

Sex (female/male)	36/28	
Handedness (right/left/ambidextrous)	59/8/1	

Age in years	8–15	10.8 (±1.8)
Test of silent reading efficiency and comprehension (TOSREC)	13–60	37.8 (±10.9)
Test of word reading efficiency (TOWRE)	64–169	124.2 (±21.3)
Non-verbal intelligence and reasoning (KBIT)	17–44	35.5 (±5.2)

### 2.3 Stimuli

**Fig. 1:**
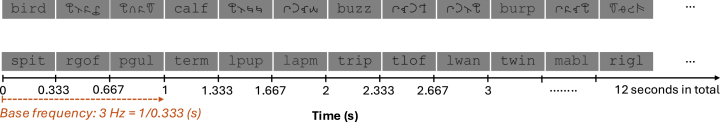
Examples of stimuli. Two trial examples illustrate the critical properties of the study, in which streams of word form stimuli constructed from a range of elements (letters, unfamiliar pseudo-characters) and configurations (words, nonwords, pseudofont strings). Stimuli were presented centrally on the screen at a rate of exactly 3 stimuli per second, across 30 trials of 12 s each. Individual stimuli and the overall temporal properties of the trial structure were adopted directly from Wang et al. (2021), which demonstrated nearly identical amplitude responses at 3 Hz and harmonics (i.e., 6 and 9 Hz) across these variations when viewed by adults.

The experiment used a base stimulation consisting of rapidly presented character strings at a frequency of 3 Hz (one string every 333 ms, three stimuli per second). Each string contained four characters (letters or letters transformed into a pseudofont), and the stimuli were drawn from three categories: words (W), nonwords (NW), and word forms displayed in a pseudofont (PF), covering a wide range of visual and orthographic features. There were 30 exemplars for each type of stimulus, for 90 exemplars in total (see Fig. 1 for examples of stimuli, and Appendix A for a full list of stimuli). All words were common, monosyllabic, singular nouns spanning a relatively wide range of word frequencies (averaged 97.7 per million, range 3–784 per million); nonwords were generated on an item-by-item basis by semi-randomly rearranging the letters of the corresponding words, yielding letter string combinations that were statistically implausible; pseudofonts were the same words stimuli presented in Brussels Artificial Character Set font (BACS-2 Serif font, Vidal and Chetail, 2017). For further details, please refer to Wang et al. (2021), which used the same stimulus set as in the present study. While Wang et al. (2021) focused on oddball amplitudes of stimulus contrasts and found no differences in base amplitude across contrasts, the present work examines base latency. Many of the design principles for this stimulus set, as described in Wang et al. (2021), were originally adapted from an SSVEP study by a vision science group in France (Lochy et al., 2015), which matched stimuli one-to-one based on the specific distribution of visual letters.

A total of 30 trials were presented, each lasting 12 s (three stimuli presented per second), resulting in 360 s of continuous stimulation per participant (Fig. 1). The trials consisted of different stimulus pairings (W&PF, NW&PF, and W&NW), with 10 trials of each type. Trials of the same type were organized into blocks, but the blocks were completed in a randomly assigned order across participants to minimize potential order effects.

### 2.4 Experimental procedure

Prior to the EEG experiment, the procedure was explained to the participant, after which the participant performed a brief practice session.

Participants were seated 1 meter away from the computer monitor in a darkened room during their EEG session. Each condition started with a blank screen shown for a random interval of 1500–2500 ms. During this time, participants were asked to fixate on a white cross in the middle of the screen. Then, each second (aka an epoch) includes the presentation of three stimuli. Twelve successive epochs made up one trial. Ten trials with unique random orders of stimuli were presented for each condition.

In order to maintain participants’ attention throughout the experiment, a font size change detection task was used. During the recording, the participant continuously fixated on the center of the screen, and pressed a button whenever they detected that the font size of the stimulus got bigger. The font size change detection task incorporated a “staircase” mode, during which the size of font change became smaller as the accuracy increased, or became bigger when the accuracy decreased. Two size change targets occurred in a non-periodic fashion within a trial.

Overall, the entire EEG experiment took around 30 min including setup, practice, and breaks between trials.

### 2.5 EEG recording and preprocessing

EEG data were collected using 128-sensor HydroCell arrays (MagstimEGI), Electrical Geodesics NetAmp300, and NetStation 5.4.2 software. Data were acquired using Cz as reference at a sampling rate of 500 Hz, and impedances were kept below 50 kΩ. Stimuli were presented using an in-house stimulus presentation software.

Before pre-processing, the data were shifted backwards in time to account for a 60 ms delay introduced by NetAmp300/NetStation software. Recordings were filtered in NetStation using a 0.3–50 Hz bandpass filter that was applied twice to minimize power from frequencies outside the filter range. Data were then imported into the in-house signal processing software for further pre-processing.

During pre-processing, EEG data were re-sampled to 420 Hz to ensure that the frame rate of 60 Hz included an integer number of time samples. Sensors were excluded if more than 15% of samples from the sensor exceeded a 60 μV amplitude threshold. Data from these sensors were replaced by the average value from six of their nearest neighboring sensors.

The continuous EEG data were then re-referenced to average Ref. Lehmann and Skrandies (1980) and segmented into 1-s epochs. Epochs with more than 10% of data samples exceeding the noise threshold of 30 μV or any part of the sample exceeding the blink threshold of 60 μV were excluded from the analysis on a sensor-by-sensor basis. Artifact rejection was performed to remove epochs containing artifacts such as blinks or eye movements. If an epoch exceeded the peak/blink threshold in more than 7 sensors, the entire epoch would be removed in all sensors.

Recursive Least Squares (RLS) filters were then used to filter continuous EEG signals in the time domain (Tang and Norcia, 1995). The filters were tuned to each of the analysis frequencies (i.e., 3 Hz, 6 Hz, 9 Hz) and converted to complex amplitude values by means of Fourier transform. Complex-valued RLS outputs were decomposed into real and imaginary coefficients for input to the spatial filtering computations of Reliable Components Analysis (RCA).

The first and last 1-s epochs of each 12-epoch trial — each comprising three stimulus events — were excluded from further analysis. This was done to reduce the influence of transient responses associated with initial exposure to the stimuli and due to more blinking often occurring at the beginning and end of a trial. Thus, analysis was performed on 10 1-s epochs per trial.

### 2.6 Analysis of EEG data

#### 2.6.1 Reliable Components Analysis (RCA)

Reliable Components Analysis (RCA Dmochowski et al., 2012, Dmochowski et al., 2015) is a matrix decomposition technique that derives a set of physiologically interpretable *reliable components* (RCs) by maximizing trial-to-trial covariance relative to within-trial covariance. Since response phases of SSVEP are constant over repeated stimulations, RCA uses this trial-to-trial reliability to decompose the entire 128-sensor array into RCs, the activations of which reflect phase-locked activities.

Given a sensor-by-feature (i.e., real and imaginary Fourier coefficients) data matrix, RCA computes linear weightings of sensors such that the resulting projected data would exhibit the maximal Pearson Product Moment Correlation Coefficients (Pearson, 1896) across trials. Each RC is a linear combination of sensors that can be visualized as scalp topographies using a forward-model projection of the eigenvectors (spatial filter vectors) (Parra et al., 2005).

Relative to other spatial filtering approaches such as Principal Components Analysis (PCA), RCA achieves high output SNR with a lower trial count (Dmochowski et al., 2015). This is especially useful for investigations involving children since it reduces the amount of data required to observe a robust signal, thereby shortening the EEG data acquisition time.

#### 2.6.2 RCA calculation

To test brain responses to visual word form stimuli, we computed RCA at the base frequency and its harmonics. Specifically, we input the real and imaginary frequency coefficients of the first three harmonics of the base frequency (i.e, 3 Hz, 6 Hz, and 9 Hz) over the 128-sensor array. To enable a direct quantitative comparison of the three conditions in a shared component space, we computed the RCA weights over the three conditions together.

#### 2.6.3 Analysis of component-space data

To choose which RCA components to examine, we first assessed statistical significance of each component’s eigenvalue coefficient via permutation test (Supplement Figure S1). The permutation test involved generating a null distribution of eigenvalue coefficients against which to compare the observed (intact) RCA coefficients by performing the RCA on 500 versions of surrogate “phase scrambled” sensor-space EEG data. This method disrupts the across-trials covariance of the data and temporal structure of the signal while preserving autocorrelation characteristics and magnitude spectra (Prichard and Theiler, 1994). Here, the phases were randomized in the frequency domain by means of rotation matrices, as described by Wang et al. (2023). Following this, we further identified the statistical significance at each harmonic for a given significant component via Hotelling’s two-sample t^2^ tests (Victor and Mast, 1991) on distributions of real and imaginary Fourier coefficients on a per-harmonic, per-component basis. Specifically, for each RC, a 1-s epoch of component-space data contained 6 data points (3 harmonics × 2 real and imaginary Fourier coefficients). Component-space data were first averaged across 1-s epochs (10 epochs per trial, for 10 trials in total in present study) on a per-participant basis. Statistical analyses were then performed across distributions of participants. Multiple comparisons were corrected using False Discovery Rate (FDR Benjamini and Yekutieli, 2001). Twenty-seven comparisons (3 harmonics × 3 components × 3 conditions) were corrected for base analyses where RCA was computed over the data from three conditions together.

To report SSVEP amplitudes across multiple significant harmonics, we used the square root of the summed squared amplitudes (“root sum square”, RSS) across multiple harmonics to combine harmonic response amplitude (Tlumak et al., 2011). We refer to this value as the RSS amplitude, in units of μV.

#### 2.6.4 Group- and individual-level latencies of RCs

Following the statistical analyses of RCs, we derived group-level latency calculations by fitting a line through the phases at harmonics with significant responses using a linear regression method, where the slope and intercept were obtained with MATLAB’s polyfit function (Norcia et al., 2020). The slope of the line represents the response latency.

In order to examine the relationship between brain processing speed and reading abilities, latency was also calculated at the individual subject level. The high SNR provided by SSVEP facilitates this individual-level analysis. We projected each subject’s sensor-space data through the spatial filter vectors derived from group-level RC analyses, resulting in individual projected data. As with the group-level calculations, we fit a linear regression line when responses were statistically significant in at least two harmonics, with the precondition that including the third non-significant harmonic would not substantially change the slope (i.e., latency).

#### 2.6.5 Validation of individual-level latency

To confirm the reliability of our individual latency estimation method and ensure that the estimated latency represents signal rather than noise, we conducted several comparisons. First, we compared latencies at both group and individual levels to check for consistency in their range. Second, we compared individual latencies across the three conditions using paired sample t-tests. Finally, we assessed the split-half reliability to rule out potential confounds from different stimulus sets/conditions in latency calculations. Specifically, we concatenated all 30 trials (epochs/s) from the three conditions and randomly split them into two halves, each containing 15 trials—five randomly selected from each condition. We then applied the individual-level latency calculation methods described in the previous section to obtain latencies for each subject in both halves and computed the correlation between them.

#### 2.6.6 Visualization of component-space data

We visualized the RCA data in three ways. First, we present topographic maps for the spatial filters of each component. Second, we present bar plots of amplitudes (μV) across harmonics, with significant responses (according to adjusted $p_{FDR}$ values of Hotelling’s t^2^ tests of the complex data) indicated with asterisks. Finally, we present phase values (radians) plotted as a function of harmonic, when responses are significant for at least two harmonics, this is accompanied by a line of best fit and slope (latency estimate).

#### 2.6.7 Analysis of brain-behavior relationships

Brain-behavior analyses were then performed to assess individual variations in the relationship between latency and participants’ reading scores, biological age and non-verbal intelligence and reasoning. The reading scores analyzed were the participants’ raw scores of TOWRE and TOSREC, representing reading efficiency and reading comprehension, respectively. Non-verbal reasoning was assessed using raw scores from the KBIT. Because latency did not differ significantly between conditions, the averaged latency across conditions was used for this brain-behavior correlation. Permutation test results showed RC1 explained the most reliability (Supplement Figure S1), so only latencies of RC1 were included.

Linear correlations were calculated between individual latencies and the TOWRE, TOSREC scores, KBIT, and age. When significant correlation was found, outliers were identified using Cook’s Distance (Cook, 1977) and removed if they exceeded the CooksD 4/n threshold (where n is the total number of data points). Correlation analyses were then performed again to assess whether the significant relationship still held after removal of influential data points. As reading skills are highly correlated with age and non-verbal intelligence (Nahavandi et al., 2020), multiple linear regression analyses, with age and KBIT as covariates, were further performed to access whether latency is genuinely correlated with reading skills, independent of age and non-verbal intelligence. Similarly, multiple linear regression analyses with reading scores and KBIT as covariates were conducted to access whether latency remains correlated with age after controlling for reading scores and intelligence.

Given the well-established association between TOWRE (single word level reading fluency) and TOSREC (sentence level reading fluency and comprehension), and parallel findings linking latency to reading, we conducted mediation analyses to assess whether TOWRE mediates the relation between latency and TOSREC. A nonparametric bootstrap method with 500 simulations was used to estimate confidence intervals and p-values for the indirect effects. Standardized regression coefficients were calculated and reported to assess effect sizes.

### 2.7 Behavioral analysis

Behavioral responses for the font size change detection task served as a measure of participants’ attention during EEG recording. We conducted one-way ANOVAs separately for reaction time and accuracy to determine whether participants were highly engaged during the whole experiment.

## 3 Results

### 3.1 Participants were highly engaged during the whole experiment

Participants completed a font size change detection task as a means of sustaining attention during the EEG session. The mean and standard deviation (SD) of d-prime and reaction time across three conditions are summarized in Table 2. Separate one-way ANOVAs indicate significant condition differences in d-prime (F(2,194)=6.02, p < 0.01). Post-hoc t-tests, with p-values adjusted using the false discovery rate (FDR) procedure to control for Type I error, revealed that size-change detection tended to be better in words&nonwords stream compared to the words&pseudofont stream ($t=2.26,p_{FDR}=0.05$), while the difference between the words&pseudofont and nonwords&pseudofonts streams ($t=1.79,p_{FDR}=0.08$) did not reach statistical significance after correction. There was no significant difference across three conditions in reaction time (F(2,194)=2.14,p=0.12).

**Table 2:** d-prime and reaction time (RT) of the font size change task performance during EEG sessions for each of three stimulus streams. d-prime was computed based on the z-transformed probabilities of hits and false alarms. Values are mean(±SD).

	Words–pseudofonts	Nonwords–pseudofonts	Words–nonwords
d-prime	1.70 (±0.38)	1.58 (±0.42)	1.82 (±0.36)
RT (s)	0.62 (±0.11)	0.61 (±0.13)	0.58 (±0.10)

### 3.2 Base stimulation elicits reliable spatial topographies and latencies across multiple harmonics

**Fig. 2:**
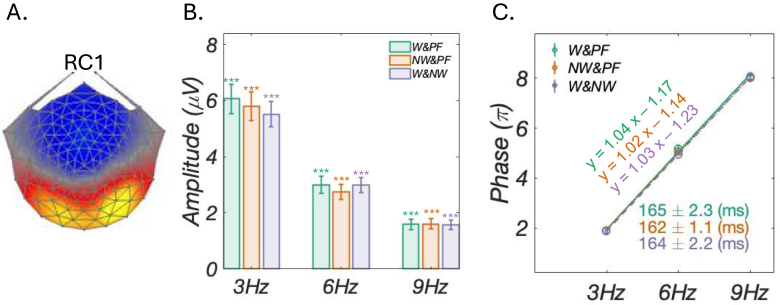
RCA analyses results. A: Topographic visualizations of the spatial filter for the first, most reliable components (RC1) across a range of word form stimulus trains; B: Amplitudes (μV) are significantly greater than zero (*: $p_{FDR}<0.05$; **: $p_{FDR}<0.01$; ***: $p_{FDR}<0.001$); C: Group-level phase (π) by harmonic frequency functions inform latency estimates in milliseconds (ms) for each of the three conditions, resulting in statistically equivalent latencies (165 ± 2.3 ms, 162 ± 1.1 ms, and 164 ± 2.2 ms, respectively).

We performed RCA on responses at 3 Hz, 6 Hz, and 9 Hz in order to investigate neural activity related to processing of category non-specific visual word features with the results being summarized in Fig. 2 for the first, most reliable component (RC1, for RCs 2 and 3, see Figure S2 in the Supplement). Fig. 2A displays topographic visualizations of the spatial filter out of RCA pooled over stimulus conditions. RC1 was distributed over bilateral occipito-temporal area, with slightly stronger responses in the right homologue. The bar plots in Fig. 2B present amplitudes of component space data at harmonics in bar plots, with statistically significant responses in all three harmonics (all $p_{FDR}<0.05$, corrected for 27 comparisons) for all three conditions. RSS amplitude comparisons across conditions showed that there is no significant difference between conditions (F(2,203)=0.05, p=0.95). The line plots of Fig. 2B display the best-fit line of phase values across three harmonics, accompanied by group-level response latencies, represented and calculated by the slope of the plot. Latencies are comparable across the three conditions (165 ± 2.2 ms, 162 ± 1.2 ms, and 164 ± 2.2 ms, respectively).

### 3.3 Individual-level latencies are highly reliable

**Fig. 3:**
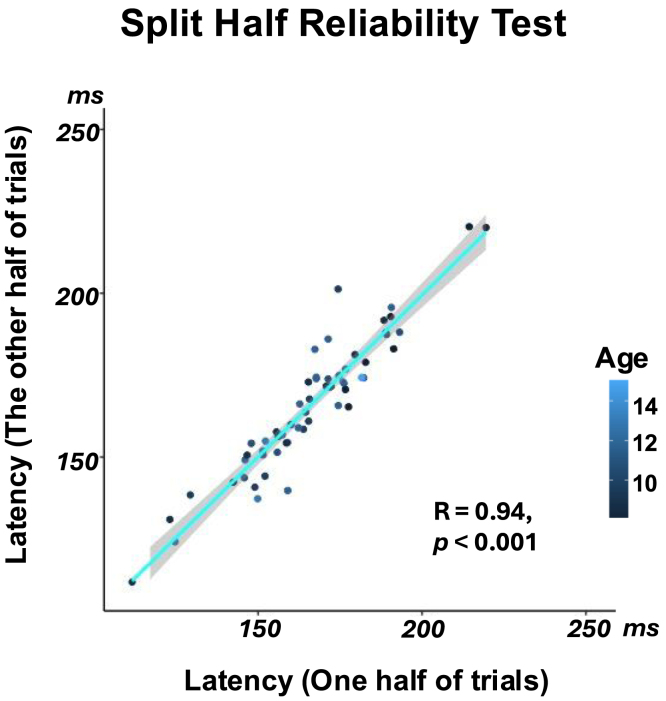
Validation of individual-level latencies. A split-half reliability test was computed by concatenating all 30 trials from the three conditions and randomly split them into two halves, each containing 15 trials—five randomly selected from each condition. R value of 0.94 demonstrates high reliability, indicating the latencies represent robust signal rather than noise, and more importantly, they are stable across variations in letter string forms.

At the individual level, 64 of the 68 participants (94%) met criteria for RC1 latency estimation (i.e., which required amplitudes significantly above zero for at least two harmonics, and a third harmonic that did not significantly impact the slope), allowing for further analyses. The group-level latencies (Supplement Figure S3.A) are broadly similar to the individual-level latencies (Supplement Figure S3.B), consistent with the well-known 170 ms findings in the ERP literature. There is no main effect of condition on the individual latencies (Supplement Figure S3.B), and individual-level latencies are significantly correlated between pairs of conditions (all p<0.001 in Supplement Figure S3.C). These findings support the most promising split-half reliability test (p<0.001 in Fig. 3D), which demonstrated high reliability of individual latency calculations across different stimulus onsets and various visual word forms.

### 3.4 Individual latencies were negatively correlated with reading scores and age

We tested brain-behavior relationships using the estimated RC1 latencies of individual subjects. We focus on response latencies only for base processing, as individual-level responses were significantly greater than zero for at least two harmonics (see Methods).

**Fig. 4:**
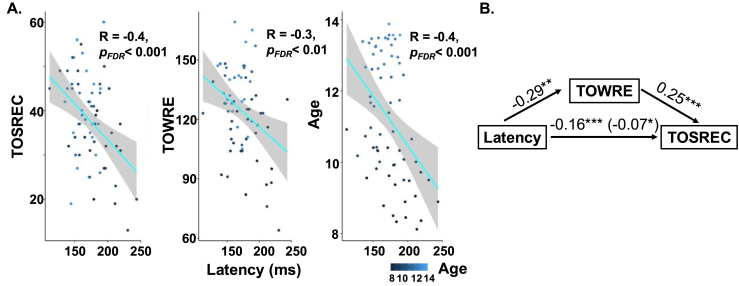
Statistically significant correlations between latency, reading skills, and age, as well as a significant mediation effect. A: Linear regressions between individual-level latency and text comprehension fluency (TOSREC, A—left), single word reading fluency (TOWRE, A—middle), and age (A—right). Children with shorter latency had better reading fluency. Multiple linear regression correlations showed that the effects of latency on reading fluency are still statistically significant when controlling for age, but the effect of sight word efficiency is not. Meantime, older children had shorter latency. This effect of age on latency remains statistically significant after controlling for reading fluency. B: The direct relationship between latency and TOSREC was significantly mediated by an indirect pathway. Specifically, latency was associated with TOWRE, which in turn affected TOSREC (see residual values in parentheses).

Cortical latency was negatively correlated with text reading fluency, word naming speed, and age (Fig. 4A). Linear correlations between latency and each reading score showed that participants with faster word naming skills (TOWRE, $r=-.4,p_{FDR}<0.01$) and more fluent text reading comprehension (TOSREC, $r=-.3,p_{FDR}<0.001$) had shorter cortical latencies. Moreover, older students had shorter latencies $r=-.4,p_{FDR}<0.001$. Multiple linear regression correlations showed that the effects of TOWRE (r=−.28,p=0.03) and TOSREC (r=−.33,p=0.01) on latency are still statistically significant when controlling for age and non-verbal intelligence. In addition, the effect of age on latency is statistically significant when controlling for reading scores and non-verbal intelligence (r=−.27,p=0.03). Furthermore, a significant negative correlation was observed between latency and non-verbal intelligence (KBIT), indicating that individuals with higher non-verbal IQ scores tended to show shorter latencies (r=−.33,p=0.008). Given the primary aim of this study was to examine the relationship between neural temporal dynamic and reading fluency, resolving the overlap between metrics of intelligence and reading comprehension fall outside the scope of this investigation.

### 3.5 Relationship between latency and sentence reading fluency is mediated by single word reading fluency

A causal mediation analysis was conducted to examine the effect of latency on sentence reading fluency (TOSREC), with single-word reading fluency (TOWRE) as the mediator. The effect of latency on TOSREC was significantly mediated via TOWRE. The standardized regression coefficient between latency and TOWRE (β=−0.29, $p_{FDR}<0.01$) and the regression coefficient between TOWRE and TOSREC (β=0.25, $p_{FDR}<0.01$) were both significant (Fig. 4B). The indirect effect was calculated as (−0.29) × (0.25) = −0.07. We tested the significance of this indirect effect using bootstrapping procedures (500 samples). The bootstrapped indirect effect was −0.07, with a 95% confidence interval of [–0.15, –0.02], and was statistically significant ($p_{FDR}=0.012$). The total effect of latency on TOSREC was also significant (β=−0.16, $p_{FDR}<0.01$), indicating that latency has both a direct and an indirect influence on text comprehension fluency.

## 4 Discussion

### 4.1 Latency calculations using SSVEP and RCA

Examining the latencies of spatial components can offer valuable insights into the temporal dynamics of cognitive operations, including speed of early cortical computations—a key factor in cognitive development. Unlike commonly used ERP paradigms that typically provide only group-level latencies and require long recording times, this study employed an SSVEP paradigm combined with an RCA spatial filtering approach. With its high signal-to-noise ratio, the SSVEP paradigm requires only 2 minutes of stimulation per condition and can provide not only group-level but, importantly, individual-level latencies using an approach that considers phase angles across multiple SSVEP harmonics (Norcia et al., 2020). Additionally, unlike conventional ERP analyses, which usually involve one or a few preselected sensors in a cluster, RCA computes data-driven weighted linear combinations across the entire montage of sensors, thereby enhancing the feasibility of obtaining the individual-level latencies and the discovery of multiple underlying brain sources. This feature is particularly advantageous in developmental research, where there is considerable inter-individual variability and potential shifts in neural sources across age groups. Furthermore, by providing latency measures, this study extends beyond the majority of previous SSVEP research, which has primarily focused on topographic analysis of response amplitude, and instead offers a dynamic, developmentally sensitive measure of reading-related neural processing.

### 4.2 Group-level latencies and brain sources underlying visual word form processing

Typically, SSVEP researchers use electrode responses at base frequencies as a baseline check, confirming that no significant differences exist between conditions before proceeding to more extensive analyses at the oddball frequency. In contrast with most previous SSVEP studies, including our own prior work that focused on the oddball response (Wang et al., 2021, Wang et al., 2023, Wang et al., 2024, Wang et al., 2025), here we closely examined the base frequency response. We find that signals at the base harmonics are more robust than those at the oddball frequencies, making them suitable for latency analysis, particularly when extended to the single-subject level. Second, using the data-driven RCA approach, we find multiple brain sources underlie base frequencies, making it promising to analyze these signals in greater detail. This is particularly significant given that the previous literature implicitly assumes that the base response is independent of stimulus category and is likely generated in primary visual areas early in the object-processing hierarchy (Lochy et al., 2016).

RCA of base frequency responses (i.e. 3 Hz and harmonics) revealed three components with differing topographies and time courses (see Figure S2). Here, we focus on the most robust component, RC1. The finding of brain responses to stimulus streams over bilateral occipito-temporal (RC1) areas aligns with many previous studies, including those with fMRI (Brem et al., 2009), ERP (Maurer et al., 2006), and SSVEP (van de Walle de Ghelcke et al., 2021, Wang et al., 2023) methodologies, which have reported preferential responses to word-like stimuli in this area. The occipito-temporal (OT) sulcus, at a site known as the *visual word form area* (VWFA) that develops with reading expertise, is particularly attuned to visual strings (Dehaene and Cohen, 2011). Importantly, the emergence of VWFA specialization is experience-dependent, shaped by learning to read and ongoing exposure to print (Saygin et al., 2016). While the VWFA is typically left-lateralized within the occipito-temporal sulcus in adults (Dehaene and Cohen, 2011), studies of developing readers have reported slightly stronger responses in the right homologue during written word processing (Maurer et al., 2006, Spironelli and Angrilli, 2009, Szaflarski et al., 2006), suggesting that both hemispheres may contribute, but their relative strength may change over development. It is possible that this slight bias for stronger signals in the right homologue reflects a difference in processing, such as an increased reliance on form-based visual word processing. Such patterns may be influenced by stimulus properties, task demands (e.g., the font-size detection task used here), or the specific occipito-temporal subregions recruited (Seghier and Price, 2011).

Phase-lag quantification of RC1 provides latencies of approximately 170 ms. This ∼170 ms latency and the OT activations are consistent with the N170 component, a neuropsychological marker of visual specialization for print, which has been consistently found in previous ERP studies comparing words or word-like stimuli with symbols (Brem et al., 2006, Maurer et al., 2006, Wang and Maurer, 2017).

Therefore, the presence of response components over occipito-temporal regions suggests that the base response is generated in cortical areas that do not yet differentiate among visual word form categories but may already distinguish broader visual categories. This indicates that object category information could be derived from the base stream, particularly under longer stimulus durations and slower presentation rates (Wang et al., 2023). Understanding how these broadly tuned responses in higher-order areas become more category-specific with age and experience is a critical question for future developmental cognitive neuroscience research.

### 4.3 Correlation of individual latencies of visual word form processing with reading and age

The possibility that higher-level orthographic processing could be derived from the base stream opens up opportunities to explore meaningful relationships between these distinctive neural responses and behavioral measures, including reading- and age-related effects.

The higher SNR of SSVEP signals, combined with RCA’s efficiency in extracting phase-locked activities, allows us to obtain highly reliable individual latencies from the majority (94%) of participants, particularly for the most robust component, RC1. This approach enables investigation of individual differences and examination of how latency varies with children’s biological age and reading abilities, providing a new direction to understand cognitive development and literacy skills.

Negative correlations were found between latencies of RC1 and reading skills, non-verbal intelligence, as well as age. Children with shorter latency had better single word (TOWRE) and sentence (TOSREC) reading fluency and comprehension, even after controlling for age and non-verbal intelligence. Additionally, older children have shorter latencies, even after reading skills and non-verbal intelligence were controlled. These results are consistent with several previous findings in the ERP literature. For example, cross-sectional comparison studies have shown how a latency shift from childhood to adulthood is attributable to increased reading skills (Amora et al., 2022, Eberhard-Moscicka et al., 2016, Maurer et al., 2011). Moreover, group comparison studies between similarly aged participants with and without reading difficulties also provide supporting evidence. For instance, N1 latencies were found to be delayed among dyslexic as compared to normal readers (Hämäläinen et al., 2011). In addition, abundant research has reported prolonged lower-level visual processing, which may affect further higher-level processing in readers with dyslexia, particularly in attention demanding (Hari and Renvall, 2001) rather than passive tasks (Regtvoort et al., 2006), presumably reflecting lower quality of automatized performance in readers with reading difficulties.

However, some ERP-based studies have found contradictory relationships between latency and reading development. For example, in school-aged children, some ERP studies have shown similar mean latencies for both typically developing children and those with reading difficulties or poor readers (Hasko et al., 2013, Kast et al., 2010, Maurer et al., 2011, Zhao et al., 2014). One study even found longer latencies in controls compared with children with reading difficulty (van Setten et al., 2019). These problematic inconsistencies in ERP-based approaches to latency also extends to the relationship between latency and age. For example, two such studies divided a school-aged sample into young and old subgroups (Maurer et al., 2011, Tong et al., 2016). Maurer et al. (2011) found a longer latency for the younger children compared to the older ones, whereas Tong et al. (2016) reported the opposite pattern. Therefore, the field is need of a new, more reliable and valid approach to investigating the relationship between latency and behavioral measures. The consistent patterns observed across all three conditions, along with robust split-half reliability, provide strong support for the reliability of this approach and its utility for future research requiring short recording durations.

### 4.4 The relationship between cortical latency and fluent reading comprehension is mediated by speed of word naming

We further examined the relationship between latency and sentence reading fluency and comprehension, and found that single word reading fluency mediates this relationship. Our findings provide evidence that latency is linked to single-word reading efficiency, which, in turn, affects sentence reading fluency and comprehension from late childhood to early adolescence. These results align with previous ERP findings showing a latency effect for print, where shorter latencies were observed for familiar single-word recognition in the native language compared to unfamiliar visual scripts from other language systems (Wang and Maurer, 2017, Wang and Maurer, 2020). Increasing processing speed for familiar visual word form processing may reflect increased automatization, driven by reading expertise developed through education and daily exposure (McCandliss et al., 2003), which in turn affects comprehension and development of text reading. This is an important finding, as it suggests that single-word reading fluency can be explicitly measured and potentially trained through tasks that engage automatic processing, even before formal reading acquisition and instruction begin. Future training studies could lay the groundwork for remediation efforts aimed at improving single-word reading efficiency, ultimately enhancing text reading fluency, comprehension, and overall literacy development.

### 4.5 Challenges and limitations

The individual-level latency estimate approach requires that the signal must meet a specific criterion: the phase-by-harmonic best-fit line must form a linear pattern of phase distribution. This constraint explains why we obtained individual-level latencies only for RC1, the most robust component, and not for other components, including those related to oddball responses.

Additionally, to examine individual differences in cortical latency, a study must include a sufficient number of participants with at least two significant harmonics (provided that including a third non-significant harmonic does not substantially alter the slope) at the individual level for meaningful across-subject comparisons.

Furthermore, although our study included both younger and older children, we did not analyze them as separate groups within each reading level. Therefore, we were unable to disentangle whether some of the observed effects were primarily due to developmental maturity or reading proficiency. Future studies could address this by explicitly comparing latency patterns across age-matched readers with different reading levels or across reading-matched children of different ages.

The stimulus streams in the current study contained mixed categories of stimuli (e.g., words, pseudofonts) rather than separate streams for each category. However, our split-half reliability test — where all trials across conditions were concatenated and randomly split into two halves — confirmed that this did not pose a problem. Additionally, the English word stimuli in the current design spanned a wide range of lexical frequencies and were randomly distributed across trials. A future design that blocks words of different frequencies (e.g., high- and medium-frequency) into separate trials could, for the first time, isolate the influence of lexical frequency on SSVEP amplitude (Wang et al., 2025). Nevertheless, future studies using pure stimulus streams for each category, including words, faces, and objects, as well as manipulating lexical properties (e.g., comparing real words across different frequency levels), would provide more precise evidence on the neurotemporal dynamics of hierarchical processing and their relationship to reading proficiency.

## 5 Conclusion

The current study examined latencies of SSVEP base stream processing, a response measure that to date has not received much attention in SSVEP research on object and text processing. Latencies for individual participants were remarkably stable across the three stimulus streams and also highly reliable, as confirmed by split-half reliability testing. Moreover, individual-participant latencies were found to correlate significantly with reading skill and age: Children who are better readers or more developmentally advanced tended to have faster pre-categorical image-level processing speed—i.e., shorter latencies, as latencies were invariant across visual word form categories, the loci of individual differences is likely upstream of lexical, and even letter form, recognition. It remains possible, however, that effects are specific to cortical pathways coarsely tuned to the category of visual word forms. These findings pave the way for future exploration of the developmental patterns of neural temporal dynamics in children with dyslexia and other reading or learning differences. Understanding these patterns may ultimately contribute to early detection efforts for children at risk of reading difficulties, potentially even prior to formal diagnosis. However, further research is needed to validate their diagnostic utility.

## Credit authorship contribution statement

**Fang Wang:** Writing – original draft, Investigation, Formal analysis, Data curation, Conceptualization. **Quynh Trang H. Nguyen:** Writing – review & editing, Investigation, Data curation, Conceptualization. **Blair Kaneshiro:** Writing – review & editing, Methodology. **Anthony M. Norcia:** Writing – review & editing. **Bruce D. McCandliss:** Writing – review & editing, Supervision, Investigation, Conceptualization.

## Ethics approval statement

The study was approved by the Institutional Review Board of Stanford University. As participants were children, a parent or legal guardian of each participant reviewed a written description of the study and gave written informed consent before the session; each participant also assented to taking part in the research.

## Declaration of competing interest

The authors declare that the research was conducted in the absence of any commercial or financial relationships that could be construed as a potential conflict of interest.

## Figures and Tables

**Figure:**
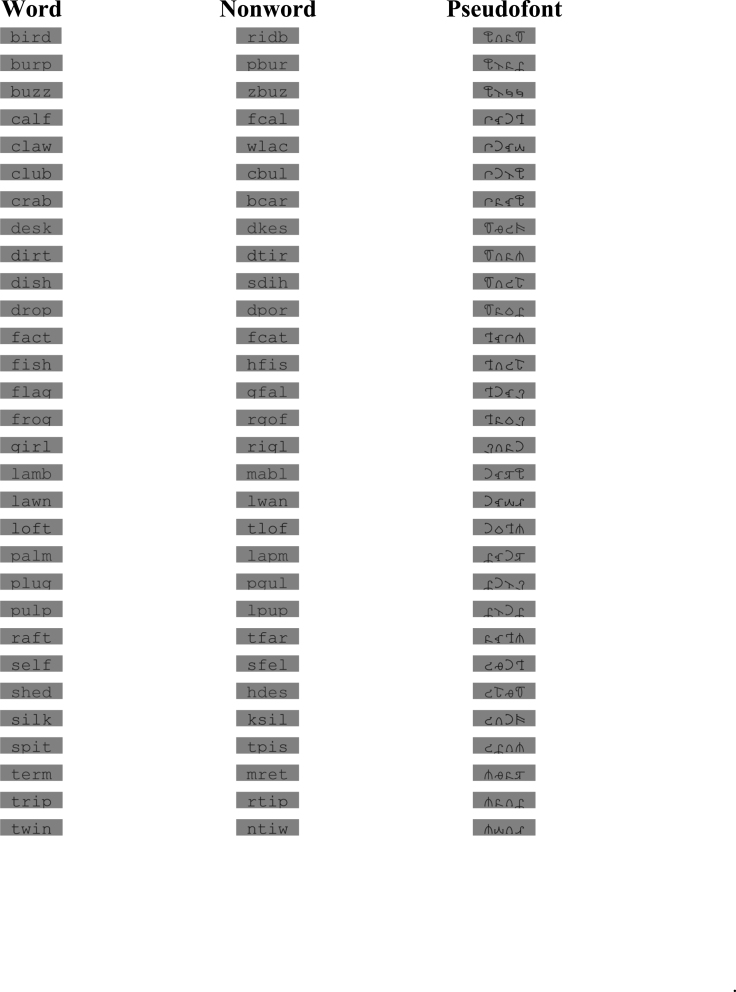


## Data Availability

The raw EEG and demographic data used in these analyses are available at: https://osf.io/gmq9a/files.

## Appendix B Supplementary data

MMC S1 Supplementary analyses of RCA components, amplitudes, and latency reliability across conditions.

## References

[b1] Álvarez-Cañizo M., Suárez-Coalla P., Cuetos F. (2015). The role of reading fluency in children’s text comprehension. Front. Psychol..

[b2] Amora K.K., Tretow A., Verwimp C., Tijms J., Leppänen P.H., Csépe V. (2022). Typical and atypical development of visual expertise for print as indexed by the visual word N1 (N170w): a systematic review. Front. Neurosci..

[b3] Benjamini Y., Yekutieli D. (2001). The control of the false discovery rate in multiple testing under dependency. Ann. Stat..

[b4] Bigozzi L., Tarchi C., Vagnoli L., Valente E., Pinto G. (2017). Reading fluency as a predictor of school outcomes across grades 4–9. Front. Psychol..

[b5] Brem S., Bucher K., Halder P., Summers P., Dietrich T., Martin E., Brandeis D. (2006). Evidence for developmental changes in the visual word processing network beyond adolescence. Neuroimage.

[b6] Brem S., Halder P., Bucher K., Summers P., Martin E., Brandeis D. (2009). Tuning of the visual word processing system: Distinct developmental ERP and fMRI effects. Hum. Brain Mapp..

[b7] Brysbaert M. (2019). How many words do we read per minute? A review and meta-analysis of reading rate. J. Mem. Lang..

[b8] Chard D.J., Vaughn S., Tyler B.-J. (2002). A synthesis of research on effective interventions for building reading fluency with elementary students with learning disabilities. J. Learn. Disabil..

[b9] Cook R.D. (1977). Detection of influential observation in linear regression. Technometrics.

[b10] Da Silva F.L., Van Rotterdam A., Van Leeuwen W.S., Tielen A. (1970). Dynamic characteristics of visual evoked potentials in the dog. II. Beta frequency selectivity in evoked potentials and background activity. Electroencephalogr. Clin. Neurophysiol..

[b11] Dehaene S., Cohen L. (2011). The unique role of the visual word form area in reading. Trends Cogn. Sci..

[b12] Dmochowski J.P., Greaves A.S., Norcia A.M. (2015). Maximally reliable spatial filtering of steady state visual evoked potentials. NeuroImage.

[b13] Dmochowski J.P., Sajda P., Dias J., Parra L.C. (2012). Correlated components of ongoing EEG point to emotionally laden attention–a possible marker of engagement?. Front. Hum. Neurosci..

[b14] Eberhard-Moscicka A.K., Jost L.B., Fehlbaum L.V., Pfenninger S.E., Maurer U. (2016). Temporal dynamics of early visual word processing–early versus late N1 sensitivity in children and adults. Neuropsychologia.

[b15] Freed E.M., Hamilton S.T., Long D.L. (2017). Comprehension in proficient readers: The nature of individual variation. J. Mem. Lang..

[b16] Hämäläinen J.A., Fosker T., Szücs D., Goswami U. (2011). N1, P2 and T-complex of the auditory brain event-related potentials to tones with varying rise times in adults with and without dyslexia. Int. J. Psychophysiol..

[b17] Hari R., Renvall H. (2001). Impaired processing of rapid stimulus sequences in dyslexia. Trends Cogn. Sci..

[b18] Hasko S., Groth K., Bruder J., Bartling J., Schulte-Körne G. (2013). The time course of reading processes in children with and without dyslexia: an ERP study. Front. Hum. Neurosci..

[b19] Hauk O., Patterson K., Woollams A., Watling L., Pulvermüller F., Rogers T.T. (2006). [Q:] When would you prefer a SOSSAGE to a SAUSAGE? [A:] At about 100 msec. ERP correlates of orthographic typicality and lexicality in written word recognition. J. Cogn. Neurosci..

[b20] Karageorgos P., Richter T., Haffmans M.-B., Schindler J., Naumann J. (2020). The role of word-recognition accuracy in the development of word-recognition speed and reading comprehension in primary school: A longitudinal examination. Cogn. Dev..

[b21] Kast M., Elmer S., Jancke L., Meyer M. (2010). ERP differences of pre-lexical processing between dyslexic and non-dyslexic children. Int. J. Psychophysiol..

[b22] Kaufman A.S. (1990).

[b23] Khanolainen D., Psyridou M., Eklund K., Aro T., Torppa M. (2024). Predicting reading fluency growth from grade 2 to age 23 with parental and child factors. Sci. Stud. Read..

[b24] Landerl K., Wimmer H. (2008). Development of word reading fluency and spelling in a consistent orthography: an 8-year follow-up. J. Educ. Psychol..

[b25] Lehmann D., Skrandies W. (1980). Reference-free identification of components of checkerboard-evoked multichannel potential fields. Electroencephalogr. Clin. Neurophysiol..

[b26] Lochy A., Van Belle G., Rossion B. (2015). A robust index of lexical representation in the left occipito-temporal cortex as evidenced by EEG responses to fast periodic visual stimulation. Neuropsychologia.

[b27] Lochy A., Van Reybroeck M., Rossion B. (2016). Left cortical specialization for visual letter strings predicts rudimentary knowledge of letter-sound association in preschoolers. Proc. Natl. Acad. Sci..

[b28] Maurer U., Brandeis D., McCandliss B.D. (2005). Fast, visual specialization for reading in English revealed by the topography of the N170 ERP response. Behav. Brain Funct..

[b29] Maurer U., Brem S., Kranz F., Bucher K., Benz R., Halder P., Brandeis D. (2006). Coarse neural tuning for print peaks when children learn to read. Neuroimage.

[b30] Maurer U., McCandliss B.D. (2007). Single-Word Reading.

[b31] Maurer U., Schulz E., Brem S., van der Mark S., Bucher K., Martin E., Brandeis D. (2011). The development of print tuning in children with dyslexia: Evidence from longitudinal ERP data supported by fMRI. Neuroimage.

[b32] McCandliss B.D. (2010). Educational neuroscience: The early years. Proc. Natl. Acad. Sci..

[b33] McCandliss B.D., Cohen L., Dehaene S. (2003). The visual word form area: expertise for reading in the fusiform gyrus. Trends Cogn. Sci..

[b34] McCandliss B.D., Posner M.I., Givon T. (1997). Brain plasticity in learning visual words. Cogn. Psychol..

[b35] McLean G.M., Stuart G.W., Coltheart V., Castles A. (2011). Visual temporal processing in dyslexia and the magnocellular deficit theory: the need for speed?. J. Exp. Psychol. [Hum. Percept.].

[b36] Nahavandi Z., Tabatabaee-Yazdi M., Samir A. (2020). Verbal and non-verbal fluid intelligence as predictors of vocabulary knowledge. J. Engl. Lang. Res..

[b37] Norcia A.M., Appelbaum L.G., Ales J.M., Cottereau B.R., Rossion B. (2015). The steady-state visual evoked potential in vision research: A review. J. Vis..

[b38] Norcia A.M., Yakovleva A., Hung B., Goldberg J.L. (2020). Dynamics of contrast decrement and increment responses in human visual cortex. Transl. Vis. Sci. Technol..

[b39] Oldfield R.C. (1971). The assessment and analysis of handedness: the Edinburgh inventory. Neuropsychologia.

[b40] Parra L.C., Spence C.D., Gerson A.D., Sajda P. (2005). Recipes for the linear analysis of EEG. NeuroImage.

[b41] Pearson K. (1896). VII. Mathematical contributions to the theory of evolution.—III. Regression, heredity, and panmixia. Philos. Trans. R. Soc. Lond. Ser. A Contain. Pap. A Math. Phys. Character.

[b42] Pennington B.F. (2006). From single to multiple deficit models of developmental disorders. Cognition.

[b43] Pikulski J.J., Chard D.J. (2005). Fluency: Bridge between decoding and reading comprehension. Read. Teach..

[b44] Posner M.I., McCandliss B.D. (1993). Converging methods for investigating lexical access. Psychological Science.

[b45] Prichard D., Theiler J. (1994). Generating surrogate data for time series with several simultaneously measured variables. Phys. Rev. Lett..

[b46] Regtvoort A.G., van Leeuwen T.H., Stoel R.D., van der Leij A. (2006). Efficiency of visual information processing in children at-risk for dyslexia: habituation of single-trial ERPs. Brain Lang..

[b47] Ruz M., Worden M.S., Tudela P., McCandliss B.D. (2005). Inattentional amnesia to words in a high attentional load task. J. Cogn. Neurosci..

[b48] Saygin Z.M., Osher D.E., Norton E.S., Youssoufian D.A., Beach S.D., Feather J., Kanwisher N. (2016). Connectivity precedes function in the development of the visual word form area. Nature Neurosci..

[b49] Seghier M.L., Price C.J. (2011). Explaining left lateralization for words in the ventral occipitotemporal cortex. J. Neurosci..

[b50] Smulders F.T. (2010). Simplifying jackknifing of ERPs and getting more out of it: Retrieving estimates of participants’ latencies. Psychophysiology.

[b51] Spironelli C., Angrilli A. (2009). Developmental aspects of automatic word processing: language lateralization of early ERP components in children, young adults and middle-aged subjects. Biol. Psychol..

[b52] Spironelli C., Penolazzi B., Vio C., Angrilli A. (2010). Cortical reorganization in dyslexic children after phonological training: evidence from early evoked potentials. Brain.

[b53] Stein J. (2023). Theories about developmental dyslexia. Brain Sci..

[b54] Szaflarski J.P., Holland S.K., Schmithorst V.J., Byars A.W. (2006). fMRI study of language lateralization in children and adults. Hum. Brain Mapp..

[b55] Tang Y., Norcia A.M. (1995). An adaptive filter for steady-state evoked responses. Electroencephalogr. Clin. Neurophysiol./Evoked Potentials Sect..

[b56] Tlumak A.I., Durrant J.D., Delgado R.E., Boston J.R. (2011). Steady-state analysis of auditory evoked potentials over a wide range of stimulus repetition rates: Profile in adults. Int. J. Audiol..

[b57] Tong X., Lo J.C.M., McBride C., Ho C.S.-h., Waye M.M.Y., Chung K.K.H., Chow B.W.-Y. (2016). Coarse and fine N1 tuning for print in younger and older Chinese children: Orthography, phonology, or semantics driven?. Neuropsychologia.

[b58] Torgesen J.K., Wagner R.K., Rashotte C.A. (2012).

[b59] van de Walle de Ghelcke A., Rossion B., Schiltz C., Lochy A. (2021). Developmental changes in neural letter-selectivity: A 1-year follow-up of beginning readers. Dev. Sci..

[b60] van Setten E.R., Maurits N.M., Maassen B.A. (2019). N1 lateralization and dyslexia: An event-related potential study in children with a familial risk of dyslexia. Dyslexia.

[b61] Victor J.D., Mast J. (1991). A new statistic for steady-state evoked potentials. Electroencephalogr. Clin. Neurophysiol..

[b62] Vidal C., Chetail F. (2017). BACS: The Brussels artificial character sets for studies in cognitive psychology and neuroscience. Behav. Res. Methods.

[b63] Volfart A., Rice G.E., Ralph M.A.L., Rossion B. (2021). Implicit, automatic semantic word categorisation in the left occipito-temporal cortex as revealed by fast periodic visual stimulation. NeuroImage.

[b64] Wagner R.K., Torgesen J.K., Rashotte C.A., Pearson N.A. (2010).

[b65] Wang F., Kaneshiro B., Strauber C.B., Hasak L., Nguyen Q.T.H., Yakovleva A., McCandliss B.D. (2021). Distinct neural sources underlying visual word form processing as revealed by steady state visual evoked potentials (SSVEP). Sci. Rep..

[b66] Wang F., Kaneshiro B., Toomarian E.Y., Gosavi R.S., Hasak L.R., Moron S., McCandliss B.D. (2024). Progress in elementary school reading linked to growth of cortical responses to familiar letter combinations within visual word forms. Dev. Sci..

[b67] Wang F., Maurer U. (2017). Top-down modulation of early print-tuned neural activity in reading. Neuropsychologia.

[b68] Wang F., Maurer U. (2020). Interaction of top-down category-level expectation and bottom-up sensory input in early stages of visual-orthographic processing. Neuropsychologia.

[b69] Wang F., Nguyen Q.T.H., Kaneshiro B., Hasak L., Wang A.M., Toomarian E.Y., McCandliss B.D. (2023). Lexical and sublexical cortical tuning for print revealed by steady-state visual evoked potentials (SSVEPs) in early readers. Dev. Sci..

[b70] Wang F., Toomarian E.Y., Gosavi R.S., Kaneshiro B., Norcia A.M., McCandliss B. (2025).

[b71] Yeatman J.D., Norcia A.M. (2016). Temporal tuning of word- and face-selective cortex. J. Cogn. Neurosci..

[b72] Yildiz M., Yildirim K., Ates S., Rasinski T., Fitzgerald S., Zimmerman B. (2014). The relationship between reading fluency and reading comprehension in fifth-grade Turkish students. Int. J. Sch. Educ. Psychol..

[b73] Yoncheva Y.N., Blau V.C., Maurer U., McCandliss B.D. (2010). Attentional focus during learning impacts N170 ERP responses to an artificial script. Dev. Neuropsychol..

[b74] Yoncheva Y.N., Wise J., McCandliss B. (2015). Hemispheric specialization for visual words is shaped by attention to sublexical units during initial learning. Brain Lang..

[b75] Zhao J., Kipp K., Gaspar C., Maurer U., Weng X., Mecklinger A., Li S. (2014). Fine neural tuning for orthographic properties of words emerges early in children reading alphabetic script. J. Cogn. Neurosci..

[b76] Ziegler J.C., Perry C., Ma-Wyatt A., Ladner D., Schulte-Körne G. (2003). Developmental dyslexia in different languages: Language-specific or universal?. J. Exp. Child Psychol..

