# Supplementary material for: Cortical latency predicts reading fluency from late childhood to early adolescence
Source: Dev Cogn Neurosci. 2025 Oct 22;77:101616. doi: 10.1016/j.dcn.2025.101616 (PMC12715427; doi:10.1016/j.dcn.2025.101616)
Supplement: MMC S1 — Supplementary analyses of RCA components, amplitudes, and latency reliability across conditions. [file mmc1.pdf]

1 Supplementary Material

1.1 SI Results

1.1.1 Permutation Test Results

Permutation testing was performed on base RCA coefficients. As shown in Figure S1, RC1–3 coefficients were all statistically significant (permutation test $p_{FDR} < 0.001$, each corrected for 3 comparisons).

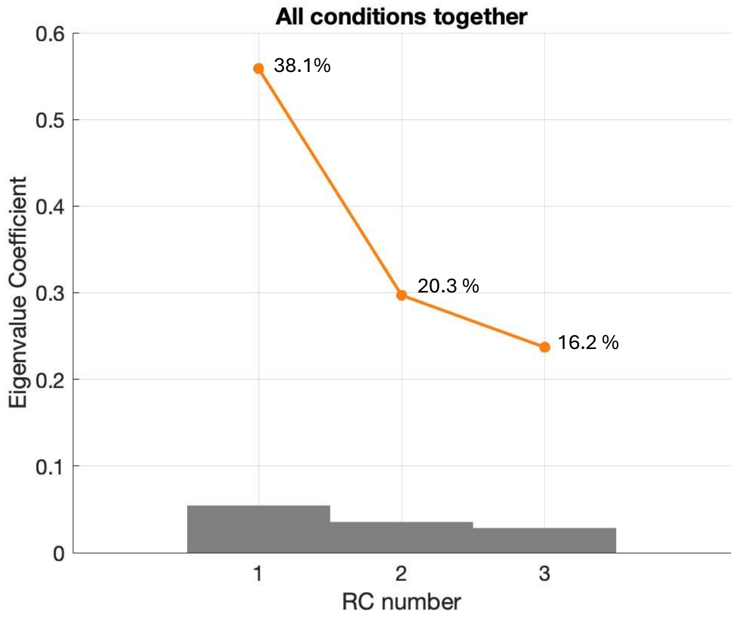

Figure S1: **Statistical analyses of eigenvalue coefficients of RC1–3.** The line plots denote the distribution of component coefficients for each RCA calculation, and the shaded gray area denotes the 95th percentile of each component's null distribution. RC1–3 coefficients for base RCA performed on three conditions together were all statistically significant (permutation test $p_{FDR} < 0.001$, each corrected for 3 comparisons). Percentage values represent the percentage of reliability explained by each RC in each condition.

1.1.2 RC2 and RC3 Results

Figure S2A displays topographic visualizations of the spatial filters out of RCA pooled over stimulus conditions for RC2 and RC3. RC2 was displaced to more temporo-parietal electrodes with right lateralization; RC3 showed maximal weightings over medial occipi-

tal areas. The bar plots in Figure S2B present amplitudes of component space data at harmonics in bar plots, with statistically significant responses in all three harmonics (all $p_{FDR} < 0.05$, corrected for 27 comparisons) for all three conditions. RSS amplitude comparisons across conditions showed that there is no significant difference between conditions (RC2: $F(2, 203) = 0.10$, $p = 0.90$; RC3: $F(2, 203) = 0.08$, $p = 0.92$). The line plots of Figure S2B display the best-fit line of phase values across three harmonics, accompanied by group-level response latencies, represented and calculated by the slope of the plot. Latencies are comparable across the three conditions for each component (RC2: 158–170 ms; RC3: 138–142 ms).

The topography of RC3 lies over early visual cortex and its 140 ms latencies may reflect the dynamics of basic visual feature processing or a very early stage of specialized orthographic processing (Schendan et al. (1998)). RC3 aligns with the P1 component observed in transient ERP studies, which occurs at around 55-170 ms in children, slightly later than that in adults at around 50-120 ms (Maurer et al. (2005)). This component is also consistent with activation of visual cortex, often the sole activation observed or focused upon in previous SSVEP studies (Lochy et al. (2015); Wang et al. (2021)).

1.2 Individual-level latencies are reliable

The group-level latencies are approximately 165 ms, as shown in Figure S3A. Individual-level latencies, which are broadly similar to the group-level latencies, are shown in Figure S3B: W-PF ($N = 63$; $m = 173$; $SEM = 3.3$); NW-PF ($N = 64$; $m = 164$; $SEM = 2.8$); W-NW ($N = 64$; $m = 167$; $SEM = 3.1$).

A one-way repeated-measures ANOVA revealed no main effect of condition on the individual latencies ($F(2, 188) = 1.41$, $p = 0.25$). Moreover, individual-level latencies are significantly correlated between pairs of conditions (all $p < 0.001$ in Figure S3C).

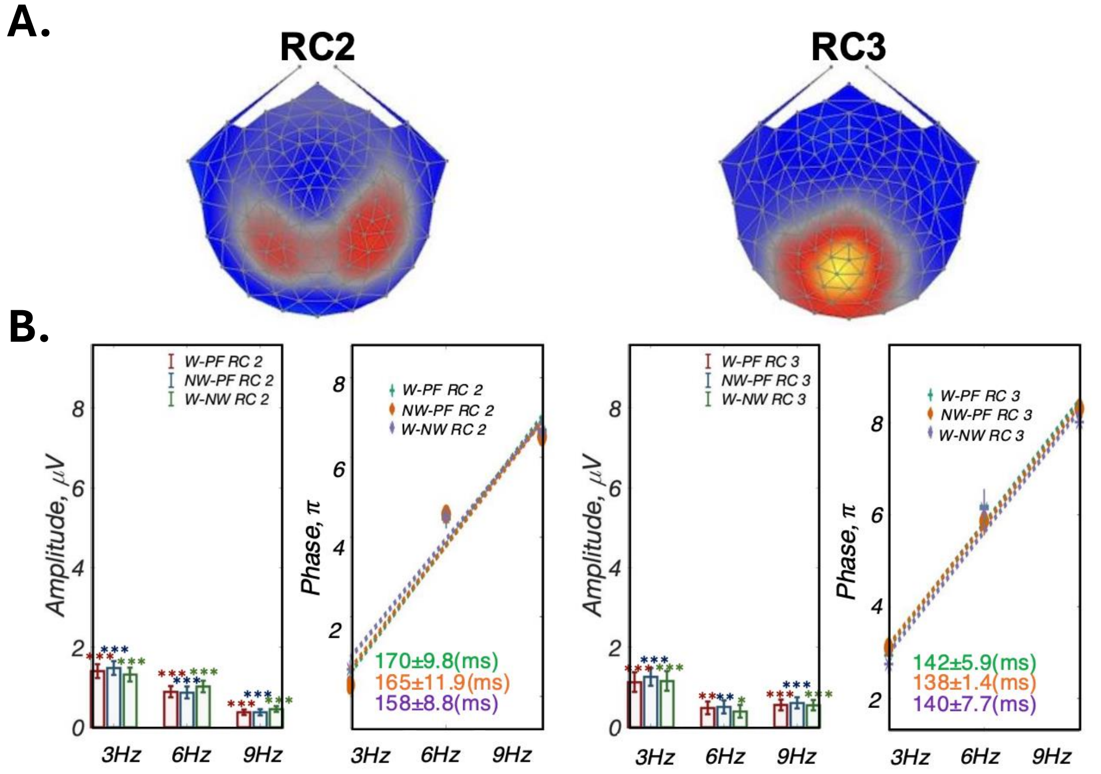

Figure S2: **Base RCA Analyses Results for RC2 and RC3.** A: Topographic visualizations of the spatial filters (RCs 2&3); B-left: Amplitude for each harmonic at each component in bar charts, all response amplitudes are statistically significant (*: $p_{FDR} < 0.05$; **: $p_{FDR} < 0.01$; ***: $p_{FDR} < 0.001$); B-right: Group-level phase and latencies of the three conditions at each component. There are no significant differences across the three conditions for any of the three components.

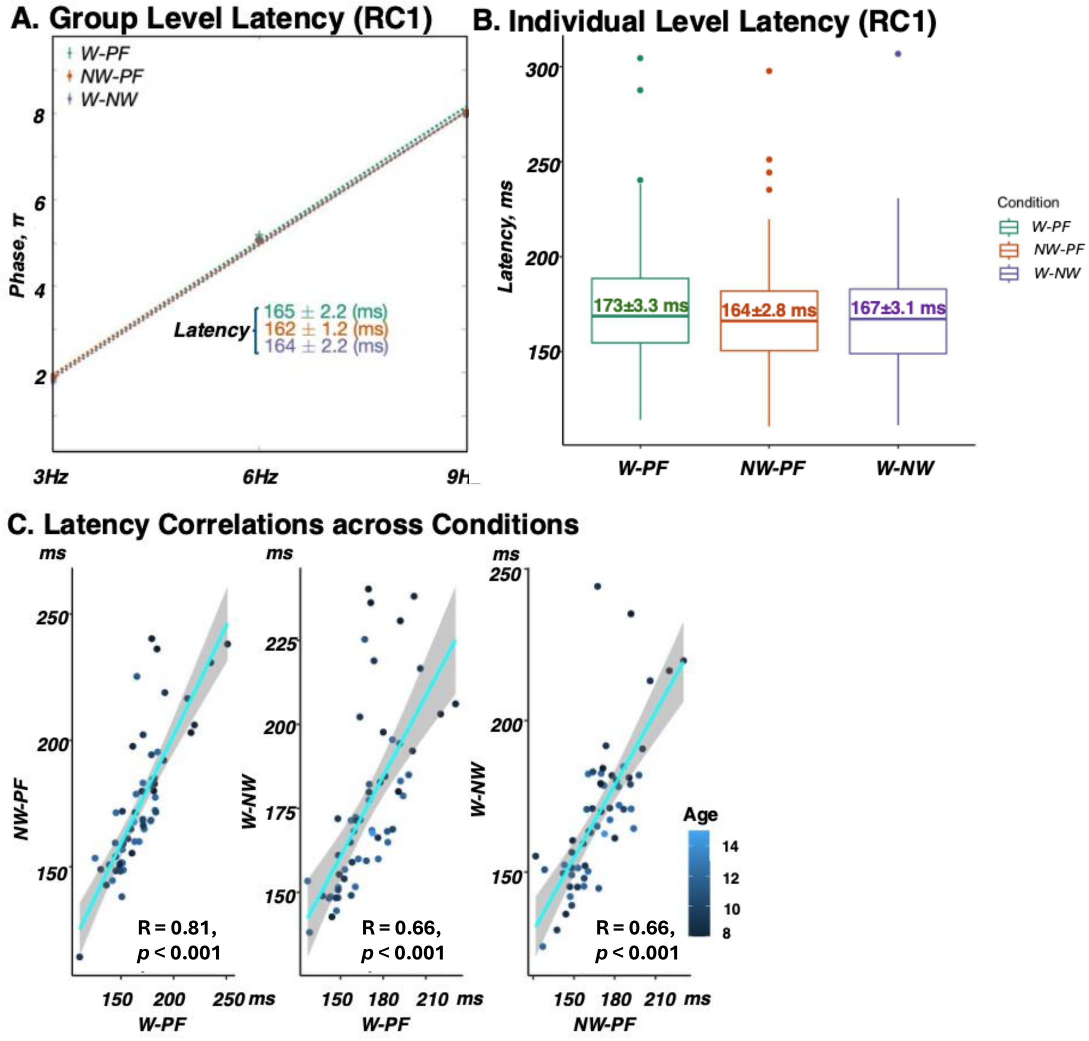

Figure S3: **Validation of individual-level latencies.** A: Group-level latencies for the three conditions are approximately 165 ms; B: Individual-level latencies across three conditions average between 160-170 ms, slightly different from the group-level latencies but still within a similar range. Individual-level latency comparisons across the three conditions show no significant condition effect ($F(2, 188) = 1.41$, $p = 0.25$); C: Latency correlations across the three conditions reveal high correlations among individual-level latencies within each condition.

References

Lochy, A., Van Belle, G., Rossion, B., 2015. A robust index of lexical representation in the left occipito-temporal cortex as evidenced by EEG responses to fast periodic visual stimulation. Neuropsychologia 66, 18–31.

Maurer, U., Brem, S., Bucher, K., Brandeis, D., 2005. Emerging neurophysiological specialization for letter strings. Journal of Cognitive Neuroscience 17, 1532–1552.

Schendan, H.E., Ganis, G., Kutas, M., 1998. Neurophysiological evidence for visual perceptual categorization of words and faces within 150 ms. Psychophysiology 35, 240–251.

Wang, F., Kaneshiro, B., Strauber, C.B., Hasak, L., Nguyen, Q.T.H., Yakovleva, A., Vildavski, V.Y., Norcia, A.M., McCandliss, B.D., 2021. Distinct neural sources underlying visual word form processing as revealed by steady state visual evoked potentials (SSVEP). Scientific reports 11, 1–15.
